# An overview of infusing service-learning in medical education

**DOI:** 10.5116/ijme.53ae.c907

**Published:** 2014-08-04

**Authors:** Trae Stewart, Zane Wubbena

**Affiliations:** Department of Counseling, Leadership, Adult Education & School Psychology, Texas State University, San Marcos, Texas, USA

**Keywords:** Service-learning, medical education, experiential learning, community service, curriculum development

## Abstract

**Objectives:**

To identify and review existing empirical research about service-learning and medical education and then to develop a framework for infusing service-learning in Doctor of Medicine or Doctor of Osteopathic Medicine curricula.

**Methods:**

We selected literature on service-learning and medical education. Articles were screened with a protocol for inclusion or exclusion at two separate stages. At stage one, articles were screened according to their titles, abstracts, and keywords. The second stage involved a full-text review. Finally, a thematic analysis using focused and selective coding was conducted.

**Results:**

Eighteen studies were analyzed spanning the years 1998 to 2012. The results from our analysis informed the development of a four-stage service-learning framework: 1) planning and preparation, 2) action, 3) reflection and demonstration, and 4) assessment and celebration.

**Conclusions:**

The presented service-learning framework can be used to develop curricula for the infusion of service-learning in medical school. Service-learning curricula in medical education have the potential to provide myriad benefits to faculty, students, community members, and university-community partnerships.

## Introduction

Medical schools are challenged to better prepare future physicians to address the increasing and complicated healthcare needs of racially and culturally diverse societies.^1^^–^^3^ As medical students transition into medical practitioners, they will encounter a growing number of patients whose health problems are the result of their environments. Impoverished communities, for example, encounter social, economic, and cultural factors that affect their health. These factors include, but are not limited to, infant mortality, asthma, obesity, mental health, drug/alcohol addictions, functional health and injuries, and children’s readiness to learn in school.^4^ In these cases, medical students must be prepared to mitigate the effects of environmental multiplicities on the health of members of a society.Correspondingly, medical school faculty must be prepared with instructional tools, strategies, and pedagogical know-how for helping future physicians understand and address the health problems encountered by members of communities in need. Unfortunately, medical school instructors’ pedagogies have changed little over time; instructor-centered, direct instruction via lectures remains the dominant pedagogy in medical education.^5^ These instructor-centered lectures reduce students to mere recipients of knowledge, which often results in student boredom and a passivity toward learning new information.^6^Pedagogies of engagement, like service-learning, empower students by providing them with an environment of authentic experiences that encourages critical thinking, problem-solving, and the application of knowledge.^6^^,^^7^ Further, experiential learning modalities contextualize knowledge beyond esoteric concepts and specialized skills learned in the classroom or laboratory. Service-learning’s central components include: “Active participation, thoughtfully organized experiences, focus on community needs and school/community coordination, academic curriculum integration, structured time for reflection, opportunities for application of skills and knowledge, extended learning opportunities, and development of a sense of caring for others”.^8^Service-learning’s focus on community makes it applicable for preparing medical students to work in communities of need. The roots of service-learning can be traced to John Dewey.^9^ Dewey focused on preventing the tendency of the student to acquire stores of knowledge useless in new situations. He noted that education of social value could not take place in the abstract (e.g., classroom, clinic). Rather, Dewey suggested that “educative [experiences] must lead out into an expanding world”.^9^^,^^10^ The community is an educative laboratory for the application of knowledge.^10^ Students can discover relationships among ideas for themselves, rather than being passive recipients of prescriptive information.Dewey’s philosophy of experience appears suited as a key pedagogical element that underpins instructional change in medical education. Medical students must understand the effects of the environment on patient wellbeing. And, medical schools can provide opportunities for students to expand their knowledge into the community through service-learning. The purpose of this study was to identify existing empirical literature on service-learning and medical education. Then, we aimed to develop an overview framework for infusing service-learning in Doctor of Medicine or Doctor of Osteopathic Medicine curricula.

## Methods

### Search strategy

A literature search was conducted for this study, and thus, no formal ethical approval was required from our university’s Institutional Review Board (IRB). First, a literature search of databases available internally through our university library were queried for the English language keywords and Boolean combinations (use of “ ” denotes the search for exact terms): “medical education” AND “service learning”; “medical education” AND “service-learning”; “medical school” AND “service-learning”; “medical school” AND “service learning”; “medical students” AND “service learning”; “medical students” AND “service-learning.” These databases were EBSCOHost, ProQuest, ERIC, JSTOR, Education Source, and dissertation/thesis abstract search engines. Then, external databases were searched: National Service-Learning Clearinghouse, Google Scholar, MEDLINE, and PubMed. Full-text PDFs of articles were saved.

### Inclusion and exclusion process

A screening protocol for the review of the articles was developed. Articles were subjected to a two-stage inclusion and exclusion screening. The first stage of the screening process required the review of each article’s title, abstract, and keywords. The inclusion criteria included empirical studies pertaining to medical students working toward a Doctor of Medicine (M.D.) or a Doctor of Osteopathic Medicine (D.O.) degree who engaged in service-learning. Articles reporting community service alone and/or service-learning in other health services (e.g., nursing, physician assistants, dentistry, pharmacy, occupational and physical therapy) were excluded because these topics exceeded the inclusion criteria parameters. Then we applied the inclusion and exclusion criteria to the full text each article at stage two of the screening process.

### Data abstraction and analysis

A thematic analysis was employed in order to inductively translate textual data into recurring themes for meaningful analysis. Our coding occurred at two levels. First, we used focused coding to develop categories and connections between central concepts.^11^ Each time a theme was identified, the text of each article was re-read. The themes included: course type, service required, participants, objectives, methods/interventions, student assessments, and results/outcomes. Then, we used selective coding for the development of subcategories related to each of the major themes identified. For example, the theme, course type, included: required, elective, and selected courses. We then synthesized the themes and subthemes together into an overview framework for infusing service-learning into medical education.

## Results

Sixty-three studies were identified using the search strategy of Boolean keyword combinations. After stage one of the inclusion and exclusion screening (i.e., review of each study’s title, abstract, and keywords), a total of 16 studies was excluded. Stage two included the full-text screening of the 47 remaining articles. After stage two, a total of 29 additional studies was excluded. The selective-sample of articles qualifying for final analysis included 18 studies spanning the years from 1998 to 2012. The inter-rater agreement for each independently reviewed full-text article was 100%. Table 1 presents summarized data from the studies that qualified for the final review.^12^^–^^29^

**Table 1:** Study characteristics of service-learning in medical school

Study	Course type	Service required	Participants	Aim or objective	Methods or intervention	Student assessment	Results or outcomes
Averill *et al.,* 2007^12^	Elective	32 hours	M1 (N = 7)	Health behavior education	Needs-assessment, health education intervention, interviews and questionnaires	Reflective essay	• Developed collaboration skills • Improved communication skills • Increased sex education knowledge • Identified community needs • Exceeded required service hrs.
Buckner *et al.,* 2010^13^	Required	1 year	M1	Health behavior education	Needs-assessment, health education intervention, presentations of outcomes, and policy recommendations for community	Reflective essay, reflective discussion, multiple-choice test, attendance	• Developed collaboration skills • Implemented health behavior interventions • Learned clinical skills related to dementia and Alzheimer’s • Addressed health-needs of underserved community • Developed teaching skills
Burrows *et al.,* 1999^14^	Required	20 hours	M1 and M2	Identify and address needs of community	Select project at school fare: clinical services, education, fund-raising, behavior interventions, obtain feedback for qualitative analysis	Hour-tracking of service, questionnaire (open-ended)	• Increased communication skills • Developed compassion, respect, and comfort working with underserved population • Learned clinical skills: taking blood pressure and testing for tuberculosis • Served as role models for youth • Exceeded volunteer hours
Elam *et al.*, 2003^15^	Elective	18 hours (each year)	M1 and M2	Health behavior education	Address community needs by using community assets	Reflective essay, questionnaire	• Identified community assets • Used community assets to address community health needs
Jefferson *et al.,* 2012^16^	Elective	1 year	M1 (N = 45)	Alzheimer’s education	Paired students with early-stage Alzheimer’s patients, conducted pretest-posttest of knowledge related to Alzheimer’s disease and dementia	Reflective essay, specialty test (Alzheimer’s disease and dementia knowledge)	• Increased communication skills • Developed clinical skills related to dementia and Alzheimer’s • Increased future likelihood of providing geriatric care • Learned about personal factors (e.g., family) affecting patients with Alzheimer’s disease
Leung *et al.,* 2007^17^	Required	6 weeks	M1 (N = 249)	Attitudes toward healthcare and knowledge of health issues	Participation in two-week training, live in underserved community while providing home-based and clinic-based healthcare	Reflective essay, reflective discussion, questionnaire	• Increased leadership skills • Increased communication skills • Developed students’ sense of social justice • No increase in critical thinking • Less confidence providing healthcare independently
Long *et al.,* 2011^18^	Elective	8 weeks (data over 5 years)	M1 (N = 62)	Leadership skills and community advocacy	Students paired with faculty mentors and community-based organizations. They completed an advocacy course, internship, and scholarly activity.	Questionnaire	• Increased leadership skills • Increased teaching skills • Developed an understanding of health disparities that could be addressed by health education interventions, community partnerships, and changes to policy/legislative mandates
McConnell *et al.,* 2010^19^	Required	1 year (data over 3 years)	M1 (N = 301)	Pediatric knowledge	Lectures on health topics, provide physical exams to students from inner-city schools	Pediatric exams, questionnaire	• Learned to identify and address the health-needs of an underserved community • Increased teaching and presentation skills
McNeal *et al.,* 2012^20^	Selective	2 to 3 months	M1, M2, M3, and M4	Health behavior education	Mini-grants awarded to student groups, groups conducted needs-assessments, held health a fare, and developed summer projects to address community needs	Poster presentations, questionnaire	• Developed leadership skills • Increased understanding of the social determinants of health • Developed understanding of health disparities that could be addressed by health education interventions, community partnerships, and changes to policy/legislative mandates
Meili *et al.,* 2011^21^	Selective	2 years	M1 and M2 (N = 14)	Develop social accountability	Service-learning program rotates through urban, rural, and international locations (students learn foreign language), qualitative data analysis conducted on open-ended questionnaires	Reflective essay	• Increased importance of patient-physician relationships • Developed multicultural understanding of community • Increased understanding of the social determinants of health • Increased sense of social justice • Recognized need to affect unhealthy habits of communities • Mixed results on students selection of medical specialty
Packer *et al.,* 2010^22^	Elective	4 days	M3 (N = 53)	Community development	Visit homeless clinics and shelters, engage in street outreach, results analyzed quantitatively and qualitatively	Reflective essay, reflective discussion, questionnaire	• Greater understanding of the legal issues and bureaucratic barriers facing healthcare • Increased sense of social justice • Exceeded volunteer hours • Mixed results on students selection of medical specialty
Sakai *et al.,* 2002^23^	Elective, Selective	1 year	M1 (N = 5)	Health behavior education	Students created curriculum, lessons on health topics at a high school, pretest-posttest design	Specialty test (sex education)	• Increased sex education knowledge • Increased teaching and presentation skills • Increased sense of social justice
Steiner *et al.,* 2000^24^	Elective	1 week	M2 (N = 71)	Natural disaster mobilization and relief	Community needs-assessments developed by not-for-profit organizations used by student groups to provide various relief efforts after a natural disaster	Reflective essay, reflective discussion, questionnaire	• Improved teamwork • Increased leadership skills • Improved communication • Identify other health-needs of underserved community • Understanding of challenges facing community organizations • Understanding of the social determinants of health problems • Understanding of communities coping with natural disasters
Stearns *et al.,* 2000^25^	Selective	1 year	M4 (N = 112)	Health factors of a rural community	Longitudinal design, students wanting to work in family healthcare located in a rural area	Poster presentation	• Identified health needs of underserved community • Increased comfort with using health-related technology • Increased understanding of limitations that affect rural community health
Switzer, 1999^26^	Selective	1 year	M1 (N = 26)	Psychosocial behavior of females who are pregnant	Pretest-posttest, Paired 26 first year med students with pregnant females who were low-income adolescents	Specialty test (prenatal care), questionnaire	• Learned clinical skills: Testing blood pressure and for tuberculosis
O’Tool *et al.,* 2005^27^	Elective, Selective	M1 (pre-clinical 8 weeks) M3 and M4 (clinical 1 month)	M1, M3, and M4 (N = 95)	Community development	Students assigned to a community organization and work with a community mentor. Students developed health projects (e.g., health needs survey, health brochures)	Reflective essay, reflective discussion, questionnaire (open-ended)	• Learned to identify and address the health-needs of an underserved community
Waddell *et al.,* 2000^28^	Required	1 year	M1 (N = 85)	Health behavior education	Identification of resources and development of plans to prevent illness and stabilize of chronic illness.	Specialty test (Genogram tool)	• Improved teamwork • Greater understanding of home-based factors affecting health
Wee *et al.,* 2011^29^	Elective	1 year (optional project swap mid-year)	M1 (N = 824)	Service-learning: home-based vs. clinic-based	Cross-comparison design between clinic-based and home-based service-learning using self-administered questionnaires	Self-administered questionnaire	• Improved teamwork • Improved communication • Developed knowledge of disease management • Greater understanding of the home-based factors affecting health • Increased understanding of the social determinants of health

Based on the review of the articles and the thematic analysis, an overview of four stages was developed for infusing service-learning in medical education. This overview was composed of the following stages: 1) planning and preparation (four subcategories); 2) action (two subcategories); 3) reflection and demonstration (one subcategory); and, 4) assessment and celebration (four subcategories).

### Planning and preparation

#### Differentiate between service-learning and community service

Service-learning encompasses a reciprocal interaction between service to the community and learning tied to specific course objectives. Conversely, service is often used in the form of episodic community-service or volunteering activities (e.g., logging community service hours to graduate; compensatory service assigned by the courts; or benefiting only students or community). One-sided activities, although beneficial in many respects, may actually detract from the potential educational benefits of service-learning when integrated into the medical education curriculum; this is especially the case when service-learning is an “add-on” to the curriculum.

#### Decide when and where to infuse service-learning into curriculum

The potential years and courses in which to infuse service-learning into the medical education curriculum depends on the course goals and objectives to be achieved. Course goals and objectives may include:

- *Society* includes a focus on the social interests of larger society and local communities;
- *Eternal and divine* sources perpetuate society by linking values, ethics, and morality;
- *Science includes* what can be observed and quantified;
- *Knowledge* of a discipline that has a particular method used to extend its boundaries; and,
- *Learners* include what we know of student’s cognition, and psychosocial development.

The selection of the goal coupled with whether the course is required and the knowledge level of participating students, informs the year of study in which students participate in service-learning. Introducing service-learning during the M1 year is important: students who had the opportunity to begin their medical education with a service-learning opportunity were more likely to carry a community-based perspective throughout their medical training, while simultaneously building a renewable pool of students/service-learners in their medical schools.^15^

### Build university-community partnerships

Effective service-learning relies on strong campus-community partnerships. These partnerships can manifest in many different variations, from institution-community, to individual course-community, and even to student organization-community. Each level of integration depends on the formality, goal, type of learning, and community-partner relationship. During the planning and preparation phase, agreements are made between the service-learning designer/instructor, service-learners/students, and the community members/organization. Students and community partners should be involved at the start to contribute to the planning process, to know what is expected, and to understand the desired outcomes from the service.The key to incorporating service-learning programs that have the ability to evolve over time is to establish relationships of trust with community-based organizations.^30^ Personal relationships and experience working together are often necessary for forming service-learning partnerships. Faculty usually work with non-profits to which they, or their colleagues, are already connected, as the trust and genuineness are usually developed already. Another approach is to build on existing campus-community partnerships for which a memorandum of understanding (MoU) exists. Many medical education programs have memoranda of understanding with community engagement or volunteer offices charged with maintaining partnerships. The community-institution partnership can too be enhanced through the co-evaluation of the service-learning program to ensure the relationship shares a common interest, complementary skills and resources, and a commitment to the instruction and assessment of medical students.Overtime, the effect of service-learning programs will contribute to building relationships of trust with community-based organizations. Relationships of trust are built though the willingness of the institution, faculty, students, and community-based organizations to develop shared values.^31^ In order to ensure that the values of the community-based organizations are aligned with the values of the participants of the service-learning program, it is important to include community partners as co-evaluators who can provide feedback on student performance.^32^ This feedback allows both students and faculty to consider the perspective of the community when determining ways to improve the service-learning program.

### Establish structure, funding, and recognition for faculty

Service-learning institutionalization requires structural (offices and policies), procedural (activities and cultural mindset), and integration. A lack of time is one pervasive explanation as to why faculty have decided not to infuse service-learning, or to stop requiring service-learning.^14^ Unlike more traditional pedagogies in which the professor prepares information to be presented to a large group of students at one time (i.e., lecture-based instruction), service-learning requires faculty to find partners, develop reflection activities and assessments to match the course objectives, juggle each student’s individual experiences, liaise with professionals at the university to create MoUs to manage risk and liability, identify readings that incorporate community health and civic elements, all while working with other faculty and dozens of students. It is understandable that faculty who must complete these additional tasks may opt-out unless provided with the necessary time and support. Dedicated time is especially crucial for the first-time implementation of service-learning. However, longitudinal service-learning projects may both decrease the overall time commitment and increase program sustainability.^33^^,^^34^Another major limitation to service-learning that affects implementation and sustainability is funding. Faculty must “sell” the concept to campus administrators by demonstrating how service is contributing to the mission, curriculum objectives, and research of the institution. A first step is to establish an engagement taskforce by including individuals familiar with community engagement and who can draft an agenda as a guide for over five years. One approach is for faculty to design service-learning programs that aim to generate funding and become self-sustainable by holding fundraising events aimed at addressing community needs.^35^ Another approach is for faculty to designing service-learning activities where students go into the community and meet with families at their home. Home-based service learning provides students with an opportunity to spend individualized time with patients while also providing students with first-hand experience of common environmental/living health concerns.^33^^,^^34^

To recognize engaged faculty, a service-learning scholar/fellows programs can be established. These competitive programs name a couple of faculty each year as “engaged scholars” who are charged with assisting their colleagues to design service-learning modules and an associated research agenda. Often, the scholars receive acknowledgement, a cash stipend, travel monies for professional development, and/or course release time for service, while the faculty who are implementing service-learning may also receive a cash stipend or course release for one semester. Another recognition event is to consider making “engagement” a theme for an academic year. A taskforce may recommend readings to faculty, invite guest speakers, hold joint campus-community activities, and host visiting fellows for trainings/workshops. Students may present their own research conducted during this time at an end-of-year poster fair.

### Action

#### Match service activity to learning goal(s)

Action is the service activity itself. Service-learning activities in medical education can be categorized into three broad types: 1) educational/training (e.g., teaching CPR to young parents, health behavior intervention programs in schools), 2) clinic-/community-based (e.g., health fair for elderly, Alzheimer patient care), 3) advocacy, policy, and outreach (e.g., Autism awareness, fundraising).^36^ Indirect (e.g., organizing first aid kits) and research projects are also service options. Regardless of type, the service must be meaningful, have academic integrity (i.e., not just thrown into a course), put the student in a position of responsibility and ownership, allow for project supervision and mentoring, place community organizations as co-instructors in student learning, and be appropriate for the needs being addressed as well as the developmental levels of the participants. Attention should be taken to ensure that the action, learning goals and objectives, and community needs are aligned.

#### Program design and student participation

The design of the service-learning course can be elective, requisite, or selection-based. From this review, service-learning elective courses are the most prominent, but often restrict participation to those students who self-selected into the course and may be, arguably, already service-oriented. Selection-based service-learning courses often require an application and acceptance in order to participate. Both elective and selection courses may provide flexibility for when student participation is limited. In order to reach all students, programs may choose to require participation in service-learning by infusing the pedagogy into the required curriculum; this allows the concept of altruism to be introduced more broadly across the diverse student population. Required service-learning is likely to be sustained over time through an ongoing volunteer of new students. In addition, sustained service-learning programs reduce the costs associated with program start-up (e.g., materials, resources, faculty training).Level of student performance also affects service-learning involvement. Brush, Markert, and Lazarus^37^ found that students in the lower and higher quartiles of class rank were less likely to participate in service-learning, while students in the middle quartiles (i.e., 66%) were more likely to engage in service-learning. Perhaps integrating a mandatory service-learning course may ameliorate performance issues of struggling students by providing them with the opportunity to learn by doing, while providing students ranked in the higher quartiles of their class with opportunities to develop communication skills by increasing personal interactions with community members. This opportunity is mutually beneficial—that is, community members can benefit from the service-learner’s expertise while the latter gains an increased understanding of the contextual problem that faced the health and wellness of an underserved community.^37^

### Reflection and demonstration

#### Best practices for organizing reflection

Reflection is the conduit between volunteer service, academic coursework, and civic intentions—the glue holding service and learning together.^38^ Reflections should be continuous (i.e., before, during, after), connected to academic and real-life needs (learning objectives for synthesizing action and thought), challenging to prompt critical thinking, contextualized within the course and service setting, and involve communication with peers, instructors, and representatives from the organization. Reflection examples include: discussion, presentations/performance, journals/writings, role-play, artwork/portfolios, research posters, and meditation.

Best practices for reflection in service-learning include: setting goals, knowing the audience, making time, choosing a method, sharing expectations, identifying resources, reviewing skills, creating transparent evaluations, demonstrating the importance of different types of reflection, and embracing/capitalizing on teachable moments. Students engaged in service-learning need to also process more than academic topics during their service experiences; additional reflection on civic engagement, stereotypes, feelings of privilege, and emotional distress is warranted. The following barriers to reflection should be avoided: reflection as an extra activity; relegating reflection toward the end of the semester; framing reflection as an individual activity or log of events; and, failing to encourage students to take a self-searching and critical stance.

#### Assessment and celebration

#### Qualitative and quantitative assessment

Assessment is necessary to determine student outcomes, document community impacts, and evaluate program structure and process, including the relationship between the community and medical institution. Quantitative measures, such as clinical-knowledge tests, attendance, and multiple-choice exams, placed outcomes in a pre-determined context. This context represented the skills, knowledge, or experiences that students were expected to have learned. A starting point for research instruments is the Compendium of Assessment and Research Tools (CART) and Project STAR. Lastly, a compendium of scales commonly used in service-learning is available in The Measure of Service Learning.^39^ These resources can help faculty locate assessment tools that are aligned with their course/program objectives.

On the other hand, qualitative measures can illuminate unexpected outcomes that may lead to greater program flexibility, if not innovation. Qualitative measures include written reflection, reflective discussion, journaling, written essays, interviews, portfolios, and open-ended questions. Poster presentations are an effective culminating activity when used to share the service-learning experience with medical school faculty, students, and community members.^40^

### Student outcomes

Student outcomes help to determine if the objectives of the service-learning activity were met. It is important to consider and report the descriptive variables (i.e., course objectives, duration, service activity, funding, community partners, participants, types of reflection, and type of assessment) in order to frame and place the results in context. Three themes of student outcomes were identified from the literature: 1) academic learning and professional development, 2) personal development, and 3) enhanced civism and social responsibility.First, the students involved in service-learning demonstrated increased academic learning and professional responsibility. This academic learning included both non-clinical and clinical skills, which often depended on the course and the student’s year in medical school when participating in service-learning. Increased self-confidence and efficacy and the development of problem analysis skills were also beneficial outcomes of students’ participation in service-learning. Further, students appeared to develop a better understanding of their roles as physicians and of professional work environments, while also developing a better understanding of public health and the impact of health legislation and policies.Next, outcomes also resulted in changes to students’ intrapersonal and interpersonal skills and leadership ability. Students immersed in the community developed their communication skills with patients, community members, other students, and school faculty. They were able to understand diversity while showing increased levels of compassion and respect for others from different backgrounds. Students also demonstrated increased teamwork, collaboration, and leadership skills. And, many became more likely to step in and take on the responsibilities associated with leadership.Lastly, students reported increased social responsibility and civism to support the underserved community. They demonstrated an increased sense of social justice and found many of the health related problems embedded in a perpetual cycle that was difficult to break. Students often found themselves eagerly engaging in more hours of service than were required. Finally, service-learners were able to support communities by helping members of these communities understand both their assets and their needs.

#### Encourage the scholarship of teaching and learning

Medical school faculty and medical school students should actively engage in scholarship aimed at reporting effective and ineffective teaching and learning practices in service-learning. The Scholarship of Teaching & Learning (SoTL) involves the study of teaching and/or learning and the public sharing and review of such work through presentations, performance, or publications.^41^ Instructors can co-write/publish with community partners and students. The publication of original scholarship in scholarly peer-reviewed journals is important to clearly articulate the methods, results, and outcomes for the evaluation and comparison of service-learning programs. Further inquiry on the topics/designs for research include: epistemological models applied to service-learning, impact of student knowledge, longitudinal/time-series, control-group designs, pretest-posttest designs, community impact studies with community groups/partners, cross-disciplinary studies connecting quantitative and qualitative outcomes, and internationally comparative studies. The improvement of service-learning in medical school is dependent on sharing examples and outcomes vis-à-vis both teaching and learning.

#### Celebrate, recognize, and share the learning

Celebration is an opportunity to acknowledge the work, learning, and appreciation of the service-learning activity. As noted in SoTL above, there is a public aspect to service-learning, which can be emphasized through special media coverage and newspaper articles written by participants, or by having everyone sign a symbolic agreement to continue the work. Students with research interests can present posters at fairs put on by the medical school or at professional academic conferences. For positive reinforcement, students can receive awards for the best poster, as decided by peers, a faculty panel, and the community partners.Technology can be used to highlight student learning, community impact, and the campus-community partnership. Students can use several free websites (e.g., Slideboom, Authorstream, YouTube) to narrate slideshows or videos to be published online. These presentations may be research-focused, descriptive, or reflective of how the experience has shaped the students’ personal and professional identities. Lastly, the Community-Campus Partnerships for Health has organized CES4Health.info, an online mechanism for peer-reviewing, publishing, and disseminating products of health-related community-engaged scholarship. Medical students may submit their presentations for possible publication. The possibility of publication may especially attract higher-ranking students to service-learning activities, since they have been found to choose engagement with research, rather than engagement with service-learning.^37^

## Discussion

The changing nature of medicine and its practice begs new approaches to educating future physicians. From this study’s review, service-learning, when infused systematically, appears to provide simultaneous opportunities to train students authentically, engage the community and university reciprocally, and develop students’ cognitive-emotional dimensions.The literature on service-learning in medical education revealed that programs across medical schools are not homogeneous. The varieties of service-learning programs reflect the multitude of community needs while also addressing the learning objectives of students at different stages of their training.A shared theme across these diverse programs, however, is that service-learning typifies authentic learning. That is, students perform real-world tasks that demonstrate meaningful application of essential knowledge and skills. Authenticity offers mastery experiences for future physicians to develop a professional identity by providing opportunities to grow in confidence, self-reliance, and self-understanding; to explore and demonstrate their abilities; and to receive supportive feedback and rewards for their actions. It permits alternatives to traditional assessment, through which educators may gain a richer understanding of their students’ strengths and challenges. For students, including authentic assessments like needs assessments and reflection activities ultimately support multiple intelligences and varied learning styles. In short, the infusion of service-learning to balance the traditional instructor-centered methods may help faculty members avoid isolating the “material of education … [that leads] to a diet of predigested materials”.^9^^,^^10^A value of engagement from service-learning is that medical students may experience transformative learning vis-à-vis cognitive-emotional development. As individuals critically reflect on their assumptions and beliefs, they can change their frames of reference, which can result in a fundamental change in the basic premises of thoughts, feelings and actions. Educators understand through information processing models that “in meaningful and sustained learning, the intellect and emotion are inseparable,” whether learning occurs at a traditional classroom or in a more informal setting.^42^ Research has suggested that pride plays a key role in the development of identity and self-esteem, especially in the context of experience of success, and may dispose students to act positively toward learning and seeking similar experiences in the future.^43^ Continued engagement by students would, therefore, advance the call for more community-centered models in which healthcare professionals adopt a population perspective and advocate for the health of the community, rather than approaching individual patients and their pathologies one symptom at a time.Medical institutions must become engaged with communities in order to collectively meet the needs and goals of all parties involved. In these “communities of practice,” engaged universities embrace communities as equal partners who work with, not for, universities in a mutual exchange to discover new knowledge and promote and apply learning.^44^ This collaborative paradigm redefines universities from curators of knowledge to dialectic partners who must reconsider how they operationalize teaching for the benefit of all—“a successful collaborative process [that] enables a group of people and organizations to combine the complementary knowledge, skills and resources so they can accomplish more together than they can on their own.”^45^In the end, a systematic approach will need to be taken in order to engage faculty, students, and the community in the benefits of service-learning. It appears through the developed framework that service-learning follows an iterative process. In such systematic processes, course content is first designed, and then the course is developed, implemented, evaluated, and revised based on the outcomes. It will be necessary to reform curriculum, prepare faculty through training, garner student participation, highlight community needs and assets through community involvement, and obtain the support at the highest levels of medical education and governmental policy. Infusing service-learning in medical school will depend on the dynamic interplay of these factors. The proposed research-based framework should feasibly guide those wanting to move toward these goals.

### Implications and future directions

This study promises immediate and long-term implications for medical education. First, for those institutions interested in infusing service-learning into its curricula, faculty training is paramount. In addition to faculty learning how to design, implement, and assess service-learning, it is equally important for faculty to use these opportunities to reflect on how their own practices can change to meet the new professional expectations of their students. Further, faculty, students, and community partners must understand the conceptual differences between service-learning and community service. Institutions can quickly support faculty development by providing financial and material resources. Recognition of those engaged in pedagogies of engagement may symbolically lend institutional support.More long-term directions will be reimagining goals and how to achieve them, including developing assessments to match and complement traditional approaches. Central to this task will be finding additional community partners/sites, perhaps utilizing a snowball sampling approach. International partnerships via study abroad or disaster assistance also appear to be promising transformative collaborative opportunities.Lastly, this review offers medical education faculty key avenues to investigate as part of their research agendas. With this in mind, as additional studies are conducted on service-learning in medical education, the literature base on which frameworks and recommendations will be broadened. Therefore, future attention should be placed on revisiting the literature as it is published, within a realistic timeframe (e.g., 3-5 years). Furthermore, as a result of discordant studies in the literature, we recommend that future research conducted on service-learning in medical education provide consistent reporting of descriptive variables (i.e., course type, course objectives, duration, service activity, funding, community partners, participants, types of reflection, and type of assessment).

### Limitations

Future research should take into account the following limitations. First, accuracy in the selection of literature may be limited by databases’ and/or journals’ poorly assigned keywords on which the search was conducted. Second, given that this study is restricted by cost and timeframe concerns, it was impossible to conduct an exhaustive search of all databases. Rather, a focused search of key databases used predominantly in service-learning and medical education was completed. Third, not all relevant manuscripts were obtainable either through lack of availability or database changes. Nonetheless, article selection was outlined in the method’s search criteria and manuscript selection and exclusion procedure.With this in mind, a fourth limitation is that researcher judgment was made about which texts to ultimately include. The process for these decisions is transparent and outlined in the methods. As is true of all studies of this type, authors’ interpretation of the results is their own construction of knowledge, based on the merging of the objective and the subjective epistemological foundations. This is further complicated when analyses are required across a pool of discordant studies, diverse in their design, methods, quality, participants, and conceptual frameworks. Our conclusions assume the included studies were previously subjected to rigorous review standards and that they are, in fact, methodologically adequate.Lastly, the literature on service-learning in medical education stems significantly from the United States. What we offer here, therefore, is an attempt to provide “generic” findings and themes for international application. However, we acknowledge that uniqueness is the norm and generalization is impossible in reality.

## Conclusion

The purpose of this study was to identify existing empirical literature on service-learning and medical education in order to develop a general framework that faculty, staff, students, and community members can use when developing service-learning to be infused into medical school curricula. A four-stage framework guides the infusion and implementation across varying medical schools’ structures, practices, and locations. Service-learning implemented in medical education has the potential to strengthen campus-community relations, improve the lives of the underserved, and develop future medical professionals who are skilled at addressing and preventing health problems interwoven into the fabric of communities in need.^46^Levine argued that new ideas in higher education would inevitably meet four fates: enclaved, diffused, re-socialized, or terminated.^47^ The determination is made by the individual and collective assessment of the faculty and leadership regarding the compatibility of the new idea with current organizational values and conditions. For this reason, and to advocate for enclaved service-learning within medical education, the proposed four-stage framework is purposefully broad and flexible to help medical schools develop an understanding of, intentional design for, implementation of, and assessment for institutionalizing service-learning that is connected to their mission statement. The components of this framework also serve to help faculty mobilize emergency service-learning projects that manifest outside of the standard curricular parameters and academic timeframes. Engagement in communities and partnerships between institutions and communities are unique, and this four-stage framework should assist medical education programs and community organizations to springboard into service-learning, or continue their momentum toward its institutionalization and refinement for students in all years of their medical career preparation.

### Conflict of Interest

The authors declare that they have no conflict of interest.
